# Gene expression plasticity under multiple stresses drives higher tolerance to a macrolide in saline and warmer environments

**DOI:** 10.1038/s44259-026-00214-7

**Published:** 2026-05-16

**Authors:** Marie Rescan, Marc Dachs Rojo, Carles M. Borrego

**Affiliations:** 1 Catalan Institute for Water Research (ICRA), Scientific and Technological Park of the University of Girona, Girona, Spain; 2 Group of Molecular Microbial Ecology, University of Girona, Girona, Spain

**Keywords:** Ecology, Ecology, Microbiology, Molecular biology

## Abstract

The widespread presence of antibiotics in the environment at sub-inhibitory concentrations imposes a selective pressure that promotes the spread of resistance. In the field, antibiotics interact with diverse physicochemical parameters that can attenuate or intensify their fitness effects. Gene expression is a central plastic trait that governs phenotypes at a higher level of integration and modulates the strength of selection, yet how synergistic or antagonistic fitness effects arise from interactions among transcriptional responses remains poorly understood. Here, we characterized gene-expression interactions underlying fitness-level interactions previously identified between a macrolide, temperature and salinity, and proposed a general methodological framework for assessing the impact of multiple stressors on gene expression. We analyzed the transcriptional response of *Escherichia coli* to azithromycin (AZI) across two salinity and temperature conditions. De novo and antagonistic interactions were prevalent, with evidence of cross-regulations between salt and AZI. High salinity increased tolerance by two orders of magnitude and, similarly to AZI, induced a downregulation of carbon metabolism. Reduced temperature, which canceled the salinity protective effect, enhanced carbon metabolism and counteracted this shift. Salinity additionally restored stress-response pathways, largely repressed by AZI. Third-order interactions attenuated the contribution of salinity relative to AZI, but the number of affected genes declined exponentially with interaction order, suggesting that higher-order interactions at the gene-expression level should play a minor role in the responses to multiple stressors. By modulating transcriptional responses to AZI, simple environmental parameters could reshape the adaptive landscape of antibiotic resistance, potentially altering the spectrum of resistance mutations likely to spread.

## Introduction

Antibiotics are now ubiquitous at sub-lethal concentrations in most, if not all, surface waters^1,2^, where they co-occur with a range of fluctuating biotic and abiotic factors such as competitive species, heavy metals, temperature or salinity. Beyond their individual effects, antibiotics and environmental variables can interact non-additively^3–7^, reducing our ability to predict their demographic consequences on microbial communities. By modulating bacterial tolerance to antibiotics, such interactions also alter the relative fitness of strains carrying antibiotic resistance genes (ARGs), likely affecting the evolutionary dynamics of resistance. Understanding bacterial demographic and evolutionary response to the widespread presence of antibiotics in the environment requires gaining insights into the mechanisms underlying multidimensional environmental tolerance.

Many genes are plastic, exhibiting variable expression levels in response to environmental cues^8,9^ and allowing for phenotypic adjustment. Yet, the broader role of the interactions at the level of gene expression remains poorly understood. Most studies considering the effect of multiple stressors on gene expression limited their analyses to each stressor’s individual effects^10–16^. In bacteria, it has been shown that antibiotics inducing a heat-shock-like response were less deleterious at higher temperatures, and vice versa^17,18^. It thus suggests that two stressors eliciting similar gene expression responses tend to interact antagonistically, resulting in combined effects that are less than the sum of individual ones. Conversely, stressors that induce opposing expression responses seem more likely to have a synergetic effect on expression.

Very little is known, however, on the distribution of interactions at the gene expression level, especially whether these interactions generally amplify or buffer the individual effects of stressors, or can even restore expression levels affected by one single or two stressors^19,20^. Few studies have concluded that combined stressors act like a totally novel stress, with interactions emerging de novo in genes that were not responsive to individual stressors^21,22^. But none explored if higher-order interactions also occur at the transcriptomic level, nor investigated the role of the regulation network on the response to multiple stressors. Can synergy and antagonism at the fitness level result from changes in the expression of a regulator in response to another stress?

We addressed these fundamental questions in the pressing context of environmental antibiotic resistance. Azithromycin (hereafter AZI) is a macrolide frequently measured in wastewater^23^, that blocks translocation during protein synthesis and, at high concentrations, leads to the toxic accumulation of misfolded proteins^24,25^. Pairwise and third-order interactions among AZI, temperature and salinity could alter by up to 100-fold the antibiotic concentration required to halve bacterial growth rate or yield^26^, which was not attributable to differential degradation of AZI. While temperature and salinity may influence AZI activity directly by altering its affinity for the translation machinery, these environmental variables more likely modulate gene expression patterns affecting cell wall and membrane permeability to AZI, clearance of misfolded proteins, or the level of toxic stress resulting from their accumulation.

We quantified the distribution of pairwise and third-order interactions between these environmental variables, namely: salinity, temperature, and AZI, on the transcriptomic response of AZI-sensitive *E. coli* populations. We provided a new classification framework for the interactions at the gene-expression level, linked them to the higher tolerance to AZI in saline and warmer environments, and discussed their potential consequences on the evolutionary dynamics of resistance.

## Results

### Salinity and higher temperature protected *E. coli* growth against AZI

**Fig. 1:**
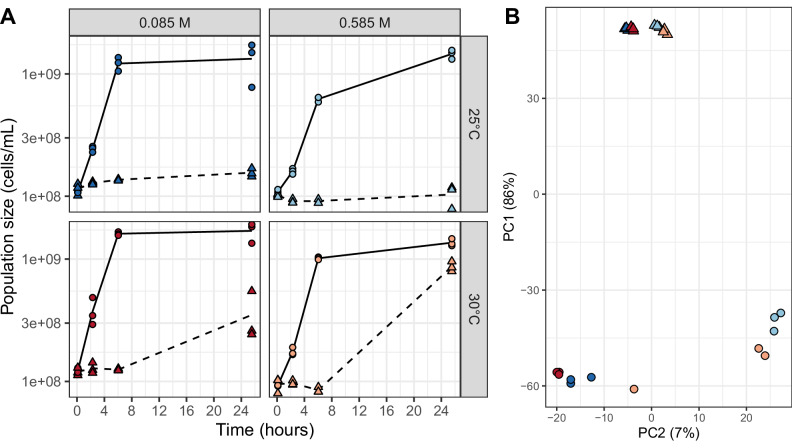
Population dynamics and transcriptomics response to combined stressors. **A** *E. coli* growth after exposure to two temperatures (rows, blue vs. red), two salinities (columns, light vs. dark colors) and exposure to none (plain lines and dots) or 1 µg mL^−1^ (dashed lines and triangles) of AZI. **B** Principal component analysis (PCA) of gene expression level for *E. coli* 6 h after exposure to the combined treatments.

Populations of *E. coli* were exposed to all cross-combinations of two salinities (0.085 M and 0.585 M), two temperatures (30 °C and 25 °C) and two concentrations of AZI (absence or a concentration around MIC of 1 µg mL^−1^), and population sizes were monitored using flow cytometry (Fig. 1A). We confirmed that high salinity reduced *E. coli* growth in the absence of AZI but offered protection against this antibiotic, while lower temperatures decreased both growth rate and AZI tolerance^26^. AZI degradation was negligible within one day and was similar across temperature and salinity treatments (see Supplementary Fig. 2 from Rescan et al. 2025^26^). Population size after 24 h was divided by 5.2 by AZI at 30 °C (*p* = 4.9 × 10^–7^). Lower temperature slightly intensified the effect of AZI (NS), but salinity nearly restored population size (multiplying final population size by 3.3 compared to AZI alone, *p* = 7.67 × 10^–4^). This protection disappeared at a lower temperature, as revealed by a negative third-order interaction dividing population size by 5.8 (*p* = 5.20 × 10^–4^).

To link the interactions at the fitness level to interactions at the gene expression level, we analyzed the transcriptomes of the populations 6 h after exposure. The changes in gene expression correlated well with the level of each stress. AZI was near the MIC at 25 °C in BHI (Fig. 1A) and explained the major part of the variance in gene expression patterns (~86%, PCA axis 1, Fig. 1B). *E. coli* could tolerate up to twice the salinity used here^26^, and in the absence of antibiotics, populations were clearly well-split by salinity along the second PCA axis (up to 7% of the variance). A 5 °C decrease did not represent a significant stress considering that 30 °C was already suboptimal, and the temperature treatments, although well clustered, explained only a marginal part of the variance in gene expression. Although interactions with salt and temperature importantly reduced the fitness effect of AZI, the PCA ordination did not evidence such interactions.

The individual and interaction effects of salinity, temperature and AZI on gene expression were analyzed using the DESeq2 package^27^ in R^28^, following Eq. 1.

1 $${\rm{Log}}\left({NormalizedCounts}\right) \sim {Salinity}\times {Temperature}\times {AZI}$$

with each explanatory variable treated as a factor.

Equation 1 can be developed into Eq. 2, which highlights individual effects of each environmental variable (first line), pairwise interactions (second line) and the third-order interaction (third line).

2 $$\begin{array}{c}\begin{array}{c}{\rm{Log}}\left({NormalizedCounts}\right) \sim {Salinity}+{Temperature}+{AZI}+\\ {Salinity}:{Temperature}+{Salinity}:{AZI}+{Temperature}:{AZI}+\end{array}\\ {Salinity}:{Temperature}:{AZI}\end{array}$$

Using the contrast command from Dseq2 results can then give the log fold change and adjusted *p* value induced by any combination of these effects. Gene expression response was considered significantly different for *p* values < 10^–6^ and log fold change below –2 or above 2. Full results are presented in Supplementary Table 1.

### Gene expression overlaps between AZI, temperature, and salinity

**Fig. 2:**
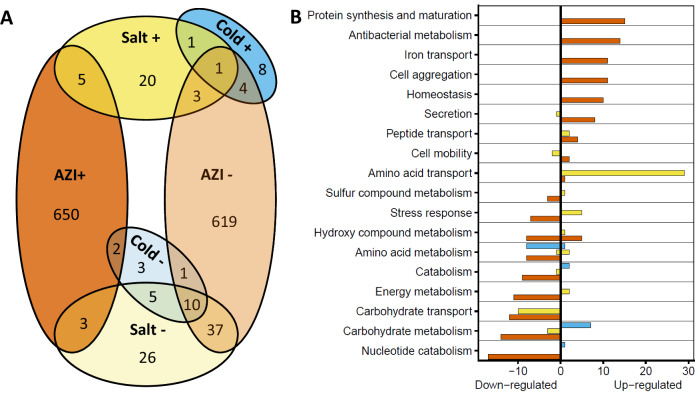
Overlaps between differentially expressed genes under experimental treatments. **A** Venn diagram displaying the number of gene transcripts under-expressed (light colors) and overexpressed (darker color) 6 h after exposure to either AZI (red), high salinity (yellow) or lower temperature (blue). **B** Number of Gene Ontology (GO) terms enriched for 17 functional groups. Colors indicate the environmental variable triggering differential gene expression (red: AZI, yellow: salt, blue: temperature).

We first analyzed differential expression induced by each treatment individually. AZI induced significant expression changes in 1335 genes (660 upregulated/675 downregulated), salinity in 110 genes (30 up/80 down), and temperature in 35 genes (14 up/21 down). All stressors produced more down- than upregulation. AZI, lower temperature, and salinity collectively induced the downregulation of the same 10 genes, with an additional 37 genes commonly downregulated by AZI and salt. By contrast, no genes were commonly upregulated by the three variables (Fig. 2A).

Differentially expressed genes were functionally annotated (Fig. 2B). AZI triggered the overexpression of pathways linked to protein synthesis, likely in response to its deleterious effect on protein folding. It also upregulated pathways related to antibacterial metabolism and iron transport, supporting the idea that antibiotic resistance can be linked to metal-tolerance mechanisms^29^. It enhanced homeostasis and upregulated cell aggregation, a strategy that reduces local antibiotic concentration. AZI tends to decrease metabolism by downregulating carbohydrate transport and metabolism, energy metabolism, and catabolism, and to shift the carbon–nitrogen metabolic balance by increasing peptide transport and protein-synthesis pathways. Unexpectedly, it repressed stress-response and sulfur-metabolism pathways, which are nevertheless protective against oxidative stress^30^.

Salinity largely upregulated pathways related to amino-acid transport. Similar to AZI, it enhanced peptide transport and downregulated carbohydrate transport and metabolism (Fig. 2B), but contrary to AZI, it upregulated stress responses and sulfur metabolism, which can buffer AZI-induced ROS^31–33^. Lower temperature, which amplified the effects of AZI (Fig. 1A), triggered the overexpression of carbohydrate metabolism, but, similar to AZI, it decreased pathways related to amino-acid metabolism.

### Antagonistic and de novo interactions acting on metabolism and stress response further explain interactions at the fitness level

In addition to the single effect of each stressor, our setup allowed us to quantify pairwise and third-order interaction effects on gene expression. For each pair of stressors, additivity was defined by a non-significant interaction at the logarithmic scale. Genes that responded neither to the individual variables nor to their interactions were not counted. About 16% of the plastic genes displayed a non-additive response to the pairs of environmental stressors, with 241 interactions found among at least one pair of stressors, which was much higher than the 71 overlaps found between at least two single variables (Fig. 2A).

Interactions between two environmental stressors can either align with or counteract the sum of their individual effects on gene expression. By considering both the direction (null, up- or downregulation) of the additive effect of isolated stress and the sign of the interaction, interactions were classified as either “de novo”, “antagonistic” or “enhancing”.

**Fig. 3:**
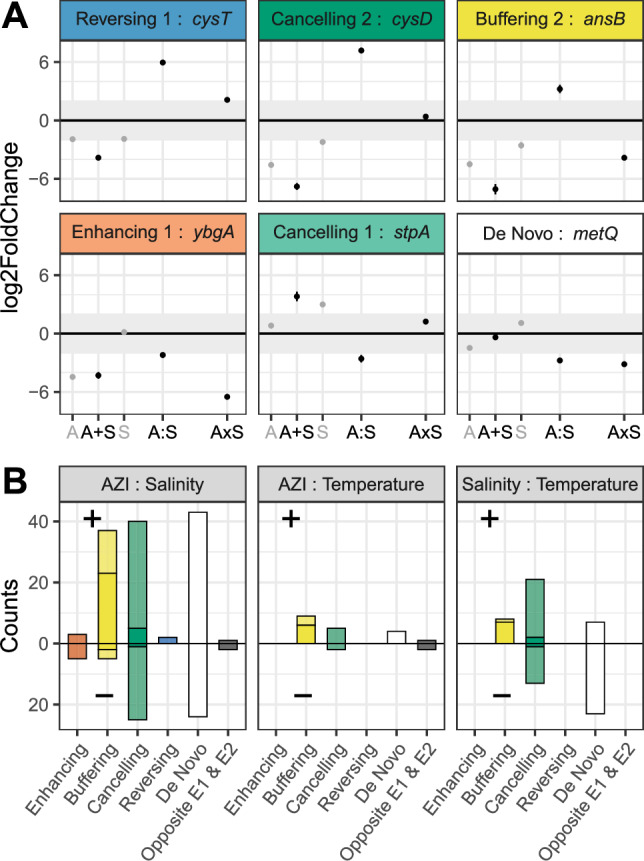
The distribution of interaction among pairs of stressors affects gene expression at the level. **A** Response of a single gene to a combination of AZI and high salinity. Interactions (positive and negative) can generate de novo effects (white), enhance (red), buffer (yellow), cancel (green), or reverse (blue) the additive effect of AZI and salt. **B** Interaction types were quantified for all combinations of pairs of stressors and split according to their regulatory effect (up or downregulation). Filling transparency shows interactions occurring when only one (transparent) or both (plain) environmental variables influenced gene expression.

De novo interactions, arising while both individual stressors had no significant effect, were surprisingly frequent (Fig. 3, white, e.g., *metQ*), and were even predominant for AZI × Salt and Salt × Temp. stressor pairs. Only a few enhancing interactions (i.e., those that increased the additive effect of two stressors) were detected, and they occurred only when one of the stressors influenced gene expression (Fig. 3, red, e.g., *ybgA*).

Many interactions were antagonistic, counteracting the effect of either one or both environmental stressors. To dig into the effects of antagonistic interactions, we considered the sign of the individual variable effects, the sign of the interaction, and the overall effect of both interacting stressors. An antagonistic interaction that did not reverse the overall direction of the combined effect relative to the sum of the individual effects was classified as “buffering” (Fig. 3, yellow). This applies, for instance, to genes upregulated by AZI and/or salinity, where a downregulating interaction occurred, but where the overall effect remained significantly positive (*e.g. ansB*). These buffering interactions were identified in 42 genes for the AZI × Salt interaction, 9 genes for AZI × Temp., and 8 genes for Temp. × Salt. Positive interactions buffering downregulation by individual variables were slightly more frequent than negative interactions, consistent with the fact that single stress triggered more down- than upregulation. Interactions that canceled out the individual effects of both stressors were classified as “canceling” interactions (Fig. 3, green, *cysD* and *stpA*). Canceling effects were slightly more frequent than buffering interactions, affecting 65 genes for AZI × Salt, 7 for AZI × Temp., and 34 for Temp. × Salt, and again, were more frequently counteracting a downregulation of the genes, generally triggered by AZI alone. In rare cases, the interaction even led to a “reversing” effect (e.g., *cysT*), where the combination of both stressors induces an overall regulation in the opposite direction compared to their individual effects (Fig. 3, blue). This occurred only in genes that were initially downregulated in response to AZI × Salt (2 genes).

Finally, only six interactions occurred between two environmental stressors that had opposite effects (gray bars in Fig. 3B), suggesting that antagonistic stressors were much less prone to interact.

**Fig. 4:**
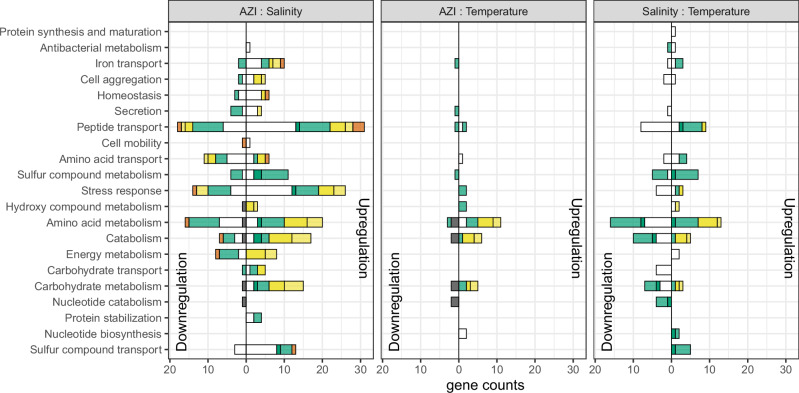
Number of genes exhibiting an interaction effect for each functional group. Colors indicate the interaction type (de novo: white, enhancing: red, buffering: yellow, canceling: green, reversing: blue), and transparency shows interactions occurring when only one (transparent) or both (plain) environmental variables alone had an effect on gene expression.

To investigate the importance of interactions between pairs of environmental stressors and their potential impact on antibiotic tolerance, each gene was assigned to at least one of the functional groups previously defined based on its associated GO terms (Fig. 4). Except for enhancing interactions, all categories were found in almost all functional groups. Canceling interactions were particularly present in pathways related to carbohydrate metabolism, catabolism and amino-acid metabolism, whereas de novo and buffering interactions were distributed evenly across functional groups.

Interactions between AZI and salt occurred in both direction in most functional groups, but pathways related to sulfur compound and iron transport, which likely play a role in AZI tolerance, were mostly upregulated. Pairwise interactions with salt also reduced AZI downregulation of metabolic pathways: catabolism, carbohydrate, energy, amino-acid and sulfur compound metabolism, and they reduced the large transport of amino-acid triggered by salt. At the functional level, de novo interactions always occur in functional groups affected by other interactions and in the same direction, further amplifying their effects.

Interactions between lower temperature and AZI were rare and mostly positive. At the functional level, these interactions increased amino-acid and carbohydrate metabolism and catabolism, counteracting a downregulation by AZI (Figs. 2 and 4). More interactions were observed between temperature and salinity than between temperature and AZI, despite the fact that AZI regulates about 30 times more genes than salinity. They mainly downregulated the catabolism, carbohydrate transport and metabolism, and buffered a downregulation of sulfur compound transport.

**Fig. 5:**
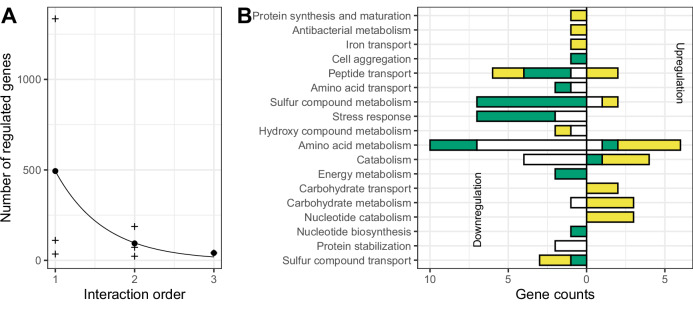
Third-order interactions. **A** Number of genes are affected by the order of the interaction. For order 1, the effect of each environment alone was considered. Crosses indicate the counts for each interaction order and each environment, while dots show the mean count for each interaction order. The line corresponds to a Poisson regression. **B** Number of genes up- (right) or down- (left) regulated by the third-order interaction in each functional semantic group. Colors indicate the interaction type (Buffering: green; canceling: yellow, de novo: white).

Twenty-one genes exhibited third-order interactions among AZI, salinity and temperature (see Supplementary Table 1 for details). We observed a near-perfect exponential decline in the average number of genes differentially expressed with increasing interaction order (*R*² > 0.98, Fig. 5.A). Although the number of stress conditions was limited, such a trend suggests that higher-order interactions ( > 3) should be rare and play only a marginal role in the response to multiple stresses. To classify the nature of the three-way interactions, we compared the predicted additive effect of individual stresses and pairwise interactions to the direction of the third-order interaction. Only de novo, buffering and canceling interactions were observed (Fig. 5B).

In many functional groups directly linked to antibiotic tolerance—antibacterial metabolism, iron transport, cell aggregation, sulfur compound metabolism, and stress response—third-order interactions buffered or canceled genes overexpression in response to individual stressors and their pairwise interactions. Third-order interaction additionally canceled the downregulation of carbohydrate transport and metabolism pathways triggered by salt and AZI, but already buffered by temperature (Figs. 2 and 4). Such downregulations reduced the effect of salinity relative to temperature, explaining its reduced protective effect at lower temperatures.

### Regulation network response to single and multiple stressors

We investigated whether the interactions observed between different environmental stressors were shaped by the gene regulatory network, and specifically, if differential expression in response to AZI could be influenced by the response of regulators to temperature or salinity. Regulator and target gene responses to every single stressor were summarized in Table 1 and multinomial logistic regressions were performed to assess the influence of regulator gene responses on the responses of their target, in reaction to the same, or a different stressor.

**Table 1:** Stressors effect on pairs of regulator-regulated genes

			**Regulator response to:**													
			AZI						Salt				Lower temp.			
			Down		0		Up		0		Up		0		Up	
			R	E	R	E	R	E	R	E	R	E	R	E	R	E
**Target response to:**	AZI	Down	**106**	**254**	451	905	**37**	**23**	594	1179	**0**	**3**	594	1180	0	2
		0	328	600	1207	2426	71	96	1601	3120	5	2	1606	3122	0	0
		Up	**87**	**88**	274	493	**37**	**24**	394	605	**4**	**0**	398	605	0	0
	Salt	Down	**7**	**63**	52	117	**7**	**1**	66	178	**0**	**3**	66	181	0	0
		0	496	864	1843	3631	137	141	2470	4634	6	2	2476	4634	0	2
		Up	**17**	**8**	32	63	**0**	**1**	46	72	**3**	**0**	49	72	0	0
	Lower temp.	Down	**4**	**15**	13	25	0	0	17	40	0	0	17	40	0	0
		0	506	911	1897	3754	143	143	2537	4803	9	5	2546	4806	0	2
		Up	**10**	**7**	16	23	0	0	26	30	0	0	26	30	0	0

For each stress, pairs of regulator-regulated genes were counted depending on the differential expression of the regulator (columns), the regulated (rows), and on the sign of the regulation (*R* for repressor, *E* for enhancer). Regulators responding to salt and temperature were only overexpressed.

Bold values indicate a significant effect of the regulator response on the target response.

Reduced temperature activated one single regulator (*metR*), but none of its two targets displayed differential expression in response to temperature. The response to salt also involved the upregulation of a single regulator, *stpA*. Genes were more likely downregulated in response to salt when they were activated by *stpA* (*p* < 3.75 × 10⁻^5^), and conversely, genes upregulated in response to salt were significantly overrepresented among those repressed by *stpA* (*p* < 1.67 × 10⁻^6^), highlighting the stabilizing role of this regulator^34^. The response to AZI involved 655 regulator-target pairs, with 47 regulators and 414 target genes both showing significant differential expression. Genes under-expression in response to AZI did not significantly depend on the response of their regulators (*p* > 0.12). Target genes upregulated in response to AZI were overrepresented among those whose repressors were also upregulated (*p* = 1.22 × 10⁻^5^) and underrepresented among those whose activators were downregulated (*p* = 2.58 × 10⁻³), suggesting a canalization of gene expression^35^. Major regulators included the global transcriptional activator *crp*, a system response repressor *arcA*, and regulators involved in stress response (*cpxR*), acid stress response (*ydeO* and *gadE*) and flagellar transcription (*flhD*). The differentially expressed target genes were typically regulated by a limited number of regulators ( ≤ 7). Among the most highly connected targets were genes encoding proteins involved in biofilm formation (*e.g. csgG*), the acid resistance transcriptional activator *gadE*, and a subunit of a multidrug efflux transporter *mdtE*.

We compared gene expression levels in response to AZI within the regulatory network under different temperature and salinity conditions. To this end, we contrasted each of the four AZI treatments against the control treatment (30 °C, minimal salinity, no AZI). Within the network of 1528 genes responsive to AZI in at least one condition, 40% of the responses qualitatively changed, between upregulated, downregulated and NS, with temperature and salinity. This ratio was 39% for the targets and 47% for the regulators, who were therefore more prone to respond to environmental variations. The regulators which differential expression in response to AZI, depending on salinity and temperature, were involved in stress response (*cpxR*), flagellar biosynthesis (*fliA*), global transcriptional regulation (*lrp* and *fis*), and stress response (*phoB* and *soxS*).

Salt and lower temperature affected respectively only one regulator, not responsive to AZI (*stpA* and *metR*). Genes upregulated by AZI were significantly overrepresented among genes inhibited by *stpA* (*p* < 4.77 × 10⁻^2^) and underrepresented among genes activated by *stpA* (*p* < 1 × 10⁻^7^), suggesting that cross-regulation participates in the antagonism between salinity and AZI. By contrast, no significant relationship was found between gene response to AZI and their regulation by *metR* (*p* = 5.56 × 10⁻²).

## Discussion

We investigated how interactions among transcriptomic responses shape fitness-level interactions between macrolides and ubiquitous environmental parameters. Expression patterns induced by AZI, temperature, and salinity in isolation provide first insights into fitness-level interactions. AZI near the MIC reprogrammed almost one-third of all genes, many linked to AZI tolerance. Antibacterial metabolism and genes involved in homeostasis and cell aggregation, which reduce local AZI concentration^36^, were enhanced. Iron transport was upregulated, consistent with cross-resistance involving metal–antibiotic efflux pumps^7,37,38^. AZI strongly reduced metabolism, and particularly repressed carbon metabolism, while redirecting amino acids toward protein synthesis.

Salinity provided a protective effect against AZ and induced a similar downregulation of carbon metabolism, while reduced temperatures, which exacerbated the detrimental effects of AZI, had an opposite effect on carbohydrate metabolism. Salinity additionally enhanced stress responses, especially sulfur metabolism, that were downregulated by AZI. H_2_S plays a well-documented protective role against oxidative stress^31,33^, such that under high salinity, sulfur-metabolism pathways can participate in antibiotic tolerance^30,32,39,40^.

Overall, 4% of all genes exhibited pairwise interactions, with 42% of the network of genes involved in AZI response displaying a different qualitative response depending on temperature and salinity. Pairwise interactions were predominantly de novo or antagonistic, often even abolishing single-stressor effects, while very few interactions amplified or reverted the effects of one or both environmental variables. AZI × temperature interactions were limited, whereas AZI × salt interactions were numerous: they upregulated stress-response genes, restored carbon metabolism, which may contribute to the protective effect of salinity. de novo interactions were the most frequent, but occurred exclusively within functional groups already altered by single stressors. At the functional level, de novo interactions tended to counteract the effects of AZI, consistent with the predominance of antagonistic interactions observed at the gene level. Very few significant interactions emerged when two environmental variables exerted opposing effects on gene expression. While interactions mostly restored baseline expression levels, it seems that when additivity alone allows one to stay close to this level, combined stress does not induce significant interactions.

Third-order interactions among AZI, salinity, and temperature affected a minor fraction of genes but tended to suppress salinity’s protective effect by downregulating antibacterial metabolism, iron transport, stress responses, and cell aggregation, while restoring carbon metabolism. This explains why salinity provided a weaker protection against the detrimental effects of AZI at lower temperatures. The number of significant interactions decreased exponentially with increasing interaction order, suggesting that higher-order interactions rapidly become negligible in the context of multiple stressors. Finally, while our conclusions are broadly generalizable, the identity and direction of antibiotic × environment interactions are likely species-specific, depending on each species’ thermal and salinity niche^26^, and should depend on the antibiotic mechanisms of action.

Our results have implications for the evolution of antibiotic resistance in the environment. ARGs' abundance differs with the salinity gradient in coastal areas^41,42^, and network analysis suggested that antibiotic resistance evolved from pathways linked to the heat and cold-shock system^17^. Environmental antibiotic concentrations are far below the MIC of most microorganisms, yet they still impose a continuous selective pressure promoting the evolution of resistance^43,44^. By modulating antibiotic tolerance, temperature, and salinity may alter the evolutionary trajectory of resistance^45^. In heavily polluted environments with sensitive bacterial populations, warmer and more saline conditions may facilitate the emergence of resistant phenotypes because enhanced tolerance enables populations to survive longer, limiting their extinction and increasing the likelihood that resistance-conferring mutations arise and spread^46^. Conversely, in urban wastewater systems, where antibiotic concentrations are far below the MIC and ARGs are continuously introduced via human waste^47,48^, higher tolerance can decrease the difference in growth rate between sensitive and resistant strains, reducing the selective advantage of resistant strains and potentially limiting their dissemination. Regulation of carbon metabolism emerges as a central factor in these interactions, and nutrient availability is likely an additional determinant of the dynamics of resistance.

Transcriptomic analyses revealed that multiple pathways contributing to AZI tolerance can be either activated or repressed depending on temperature and salinity. Because gene expression positively correlates with selection strength^49,50^, one can expect that a mutation conferring resistance should fix more rapidly under antibiotic pressure if salt, temperature, or their interaction with AZI drives its overexpression. Conversely, mutations that confer resistance but carry fitness costs in the absence of antibiotics are more likely to persist at low frequency via genetic drift if they occur in genes under-expressed and exposed to weaker purifying selection. Thus, even common environmental parameters such as temperature and salinity seem to reshape the adaptive landscape of antibiotic resistance, thereby altering not only the dynamics of resistance evolution but also the spectrum of resistance mutations that are likely to spread. Experimental evolution of antibiotic resistance under controlled conditions, accounting for environmental complexity, is the necessary next step to confirm these hypotheses.

## Methods

### Strain and environmental treatments

*E. coli* K12 MG1665 was cultured in Brain Heart Infusion (hereafter BHI) and exposed to all combinations of two salinities (0.085 M and 0.585 M NaCl), two temperatures (25 °C and 30 °C) in the presence or absence of AZI (1 µg mL^–1^). Each treatment was replicated 3 times, making 24 independent populations.

Cultures were initiated from a frozen stock, plated on BHI agar and grown for 3 days at 30 °C. One colony was then picked and inoculated into 10 mL BHI broth. After overnight growth, cultures were diluted by 1:200 into fresh BHI broth in two Falcon Tubes placed overnight at the treatment temperature (25 ° and 30 °C). Cultures were then diluted in fresh BHI to reach an OD_590_ = 0.56, and 500 µL of this pre-diluted culture was transferred into 6-well microplates filled with 6.5 mL of BHI with targeted salinity and antibiotic concentrations and incubated for 3 days.

### Population density measures

Population sizes were assessed by flow cytometry, right after inoculation and after 2 h, 6 h and 24 h. Cultures were mixed manually by carefully pipetting up and down, and 10 µL were sampled and diluted from 10^–4^ to 10^–5^ into sterile PBS, stained with the nuclear binding fluorescent dye Cystain® Green and analyzed through a Cube6^®^ flow cytometer (Sysmex, Germany). Resulting files from cytometric measurements were processed using the Floreada.io online tool (https://floreada.io/). Gating was performed based on side scatter (SSC), green fluorescence (Cystain Green) and red fluorescence (Cystain Red) to separate *E. coli* cells from noise and debris (Supplementary Fig. 1). The density of events in the gate was corrected by the dilution factor to compute population density.

### RNA extraction, library preparation and Illumina sequencing

After 6 h, microplates were vortexed, and 1.5 mL were harvested from each of the 24 cultivation wells. Culture samples were then diluted in 3 mL RNAprotect (QIAGEN, Germany), mixed for 5 s and incubated for 5 min at room temperature. The samples were centrifuged at 5000 × *g* for 20 min, the supernatant was removed, and pellets were frozen at –80 °C.

RNA was extracted and purified using the RNeasy (R) Protect Bacteria Mini Kit (QIAGEN, Germany) with a previous digestion with lysozyme and proteinase K. Briefly, pellets were lysed during 10 min with agitation in TE buffer containing 20 mg mL^–1^ proteinase K and 15 mg mL^–1^ lysozyme. The lysate was washed on RNeasy Mini spin columns with RLT containing 10 µL mL^−1^ β-mercaptoethanol. Ethanol was added, and the mix was vortexed after each step. Solutions resulting from extraction were washed twice on RNeasy Mini spin columns with buffer RW1, then RPE, and RNA was finally eluted in 50 µL RNase-free water.

RNA was quantified using the Qubit RNA Assay Kit on a Qubit 4 fluorometer (ThermoFisher Scientific^®^), and RNA integrity was assessed by electrophoresis on a 3.7% bleach agarose gel^51^ with SYBR Safe DNA gel Stain (ThermoFisher Scientific^®^). RNA quantity was lower than the sequencing threshold for two replicates of the environmental treatment at 30 °C, [NaCl] = 0.085 M and [AZI] = 0. To overcome this limitation, we split the remaining replica population for this treatment into three subsamples that were further subjected to RNA extraction as explained above. The resulting RNA extracts represent distinct pools of individuals exposed to identical environmental conditions and were considered independent biological replicates. Because any random mutation arising independently in the three populations would not have sufficient time to spread within a 6-hour period, potential differences among replicates reflect only micro-environmental variability. These differences were limited in this treatment but comparable to those observed under other treatments (Fig. 1).

RNA extracts were sent to the National Center for Genomic Analysis (CNAG, Barcelona, Spain) for library preparation with the Ribo-Zero Plus Microbiome Depletion kit for ribosomal RNA removal, and Illumina NovaSeq 6000 sequencing of 50 bp paired-end strands in two lanes.

### Read counts

Reads from the first and second lanes were merged, and sequence quality was checked using FastQC^52^ and MultiQC^53^. Trimmomatic^54^ was used for Nextera adapter removal, trimming of the 3 first three bases with medium quality, and a sliding window of five bases was used to remove all reads with an average score quality below 25 in the window. Curated reads were then aligned and mapped to the *E. coli* K12 MG1665 reference genome (NCBI GCF_904425475.1) using HiSat2^55^ with parameters optimized for bacterial genomes (non-spliced alignment and retaining up to 200 alignments per read to handle repetitive regions). SAM files were then converted to BAM format, sorted, and indexed with SamTools^56^. Read counting was performed using FADU. Raw fastq. and processed files were deposited on the NCBI Gene Expression Omnibus (GEO) repository with accession number GSE308980.

### Differential expression analysis

The following analyses were performed on R^28^, and the code was deposited in ZENODO under accession number 17493233.

Differential expression was performed using the DESeq2 package^27^. Raw counts from FADU were imported and organized into a count matrix. Metadata, including salinity, temperature and antibiotic concentration, were provided for all samples. Equation 1 in the Result section was used to test the effect of the interactions between salinity, temperature and AZI, with each explanatory variable treated as a factor. Genes were considered significantly expressed for *p* values < 10^–6^ and log fold change below –2 or above 2.

Significant interactions were then classified depending on their effect on the expression level. Each interaction occurring between two environmental variables triggering the same effect (downregulation or upregulation) or no effect for one of them, was classified as either antagonistic (opposite effect as the single variables in isolation) or synergetic (same effect). Antagonistic interactions were further classified as buffering, reversing or canceling, if the global change in gene expression was conserved, canceled or reversed compared to the additive effect of the variables in isolation. This global expression was tested by contrasting each antibiotic treatment against the control treatment (30 °C, [NaCl] = 0.085 M, and [AZI] = 0) in Dseq2 results.

### GO and semantic analysis

All differentially expressed genes were annotated using the NCBI reference GCF_904425475.1 used for mapping. Gene ontology (GO) enrichment analysis was performed to identify biological processes significantly enriched under the different treatments and *p* values obtained from the differentially expressed analysis (Eq. 1). We annotated the enriched GO terms by performing a semantic analysis. All enriched GO terms were clustered by *kmeans*. Sets of clusters were defined for 10 to 30 centers. The average silhouette width was highest for 24 centers; therefore, 24 clusters were retained for biological interpretation (a few were subsequently merged).

### Gene regulation network

*E. coli* gene regulation network was downloaded from Ecocyc^57^. Overall, 9440 interacting pairs of regulator/target were labeled as “activating” or “inhibiting” and the regulator and target response to AZI, salt and temperature and their interactions was established from the differential expression analysis (Eq. 1). Multinomial logistic regressions were performed to assess the influence of regulator gene responses on the responses of their target genes, in reaction to the same, or a different stressor. Finally, 1238 pairs of regulator/target genes, both differentially expressed under AZI in at least one temperature and salinity condition (Eq. 2), were kept for designing the AZI regulation network and its sensitivity to the environmental conditions.

## Supplementary information

Supplementary Information

Supplementary_Table_1

## Data Availability

The RNA-seq data (raw and processed files) obtained for this analysis were deposited on the NCBI GEO repository with accession number GSE308980: https://www.ncbi.nlm.nih.gov/geo/query/acc.cgi?acc=GSE308980. The code for statistical analyses and figure generation was deposited in ZENODO under accession number 17493233: https://zenodo.org/records/17493233.
