# Supplementary material for: Gene expression plasticity under multiple stresses drives higher tolerance to a macrolide in saline and warmer environments
Source: NPJ Antimicrob Resist. 2026 May 16;4:41. doi: 10.1038/s44259-026-00214-7 (PMC13190847; doi:10.1038/s44259-026-00214-7)
Supplement: Supplementary file 1 — Supplementary Information [file 44259_2026_214_MOESM1_ESM.pdf]

# Supplementary Figures

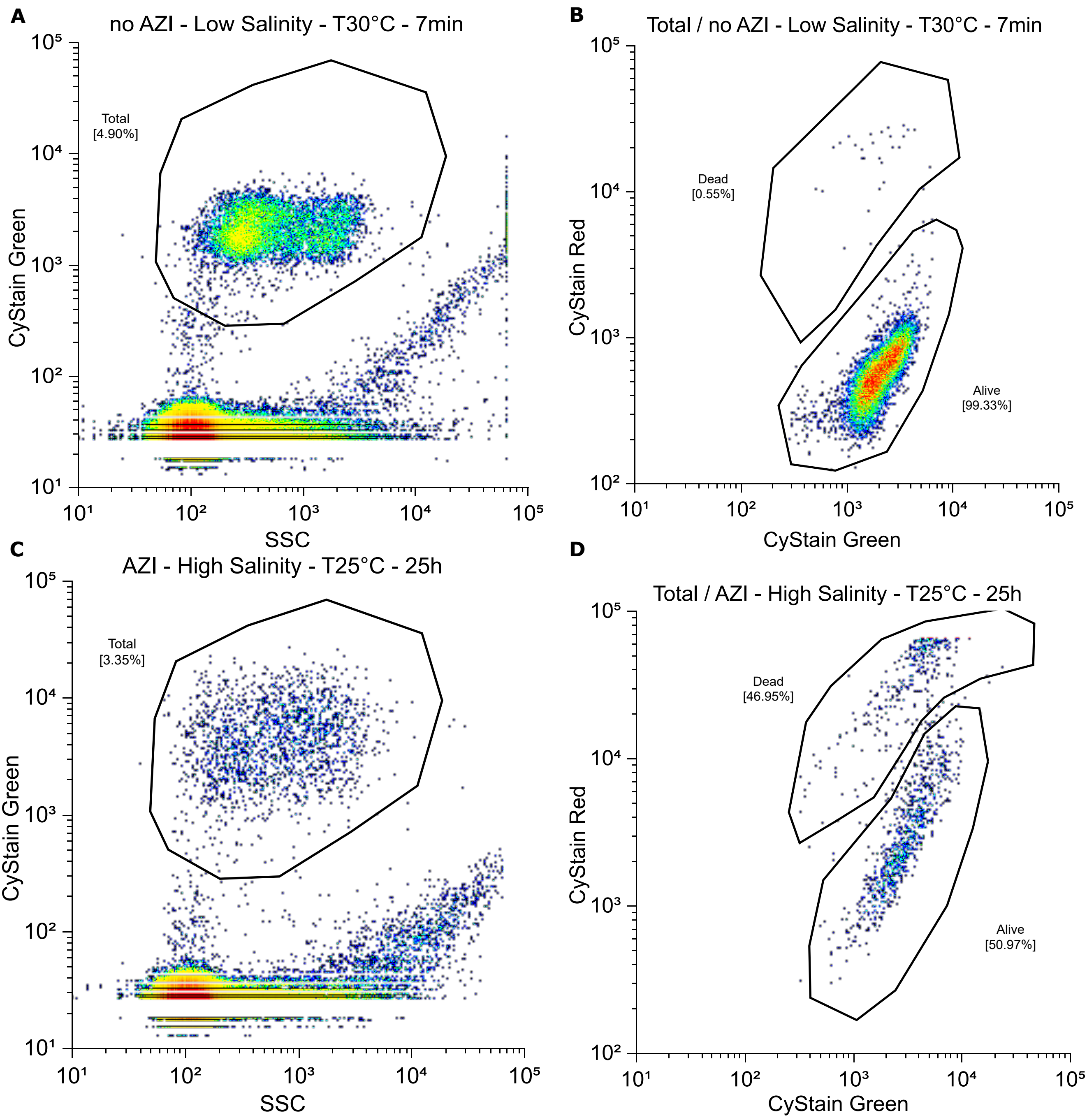


**Supplementary Figure 1: Cytometer counts of E. coli cells for two populations at different time points and under different treatments**. Gating was performed using the online tool Floreada.io (https://floreada.io/). (A, C) Total counts of E. coli cells were determined based on side scatter (SSC) and green fluorescence (Cystain Green, a membrane permeable dye, Sysmex) to separate bacterial cells from noise and debris. (B, D) Live and dead cells were distinguished based on green and red fluorescence (Cystain Red, not membrane permeable, Sysmex).

# Supplementary Tables

**Supplementary Table 1. Summary of the differential expression analysis.** Each row corresponds to one gene. Columns report the $log_2$ fold change induced by each environmental parameter or their interactions (*log2FoldChange_**), the corresponding standard error (*lfcSE_*), the Wald test–adjusted *p*-value (*padj_**), the significance of the expression change (*test_*; based on both fold-change magnitude and Wald test results), and the category of each pairwise or third-order interaction (e.g., AZI : Salinity). Here, * stands for: A = AZI, S = Salinity, and T = Temperature; A:S, A:T, S:T = interaction between parameters; AxS, AxT, SxT = total effect of two combined parameters; A+S, A+T, S+T = additive effect of two parameters; A:S:T = third-order interaction; and A+S+T+A:S+A:T+S:T = additive and pairwise effects; AxSxT = combined effect of the three parameters.
